# Epileptic spasms and RNA analysis in a new case of Kabuki syndrome type 2

**DOI:** 10.1111/epi.18362

**Published:** 2025-03-11

**Authors:** Flavia Privitera, Camilla Meossi, Filippo Maria Santorelli, Emanuele Bartolini

**Affiliations:** ^1^ Department of Developmental Neuroscience IRCCS Stella Maris Foundation Pisa Italy; ^2^ Molecular Medicine IRCCS Stella Maris Foundation Pisa Italy; ^3^ Tuscany PhD Program in Neurosciences University of Florence Italy

To the Editors,

Kabuki syndrome (KS; Online Mendelian Inheritance in Man [OMIM] #147920, #300867) is a rare genetic syndrome characterized by postnatal growth retardation, distinctive facial dysmorphisms, and mild‐to‐moderate intellectual disability (ID).^1^ Approximately 55%–75% of KS cases are caused by autosomal dominant pathogenic variants in *KMT2D* (OMIM *602113); an X‐linked inheritance has also been described, in association with variants in the *KDM6A* (OMIM *300128) gene.^2^, ^3^, ^4^ Epilepsy is often reported in KS, but the detailed clinical characteristics are often partial in the *KDM6A*‐related forms probably because of their lower general prevalence. Here, we present a 19‐year‐old girl showing severe ID, speech delay, facial dysmorphisms (Figure 1A–C), and drug‐resistant epilepsy, mostly characterized by epileptic spasms. Her clinical features were generally attributable to a chromatinopathy, and a diagnosis of Cornelia De Lange syndrome was initially entertained.

**FIGURE 1:**
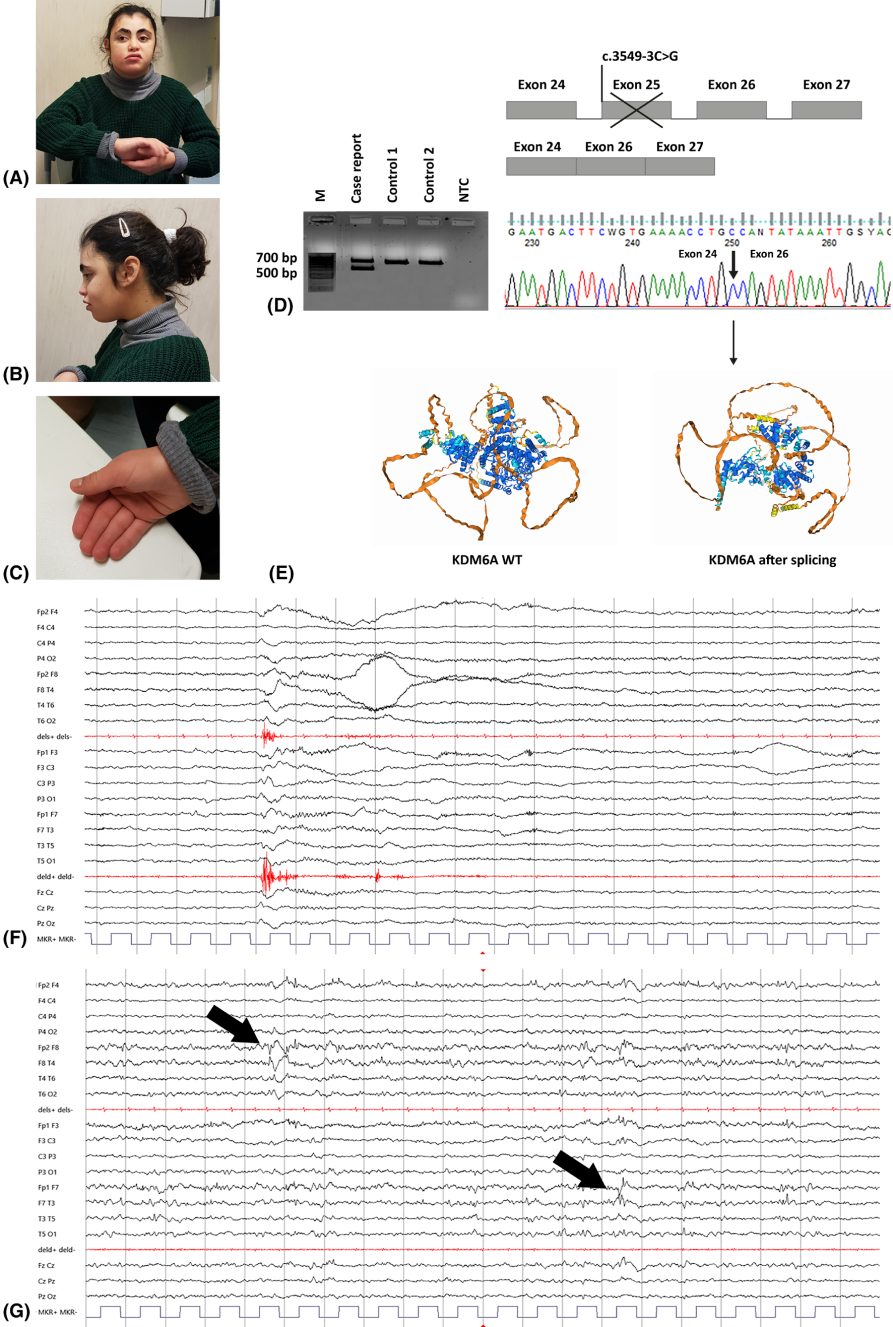
(A–C) Dysmorphic features of our patient, particularly evocative of Kabuki syndrome, including widow's peak; advanced forehead hairline; thick, arched eyebrows; bilateral eyelid ptosis; upturned palpebral fissures; long eyelashes; bulbous nasal tip; short philtrum; full lips; downturned oral fissures; and persistent fetal pads. Sialorrhea was also seen. (D) Schematic representation of the altered splicing process due to the c.3549‐3C>G variant. RNA purification and cDNA retrotranscription were performed in the index case and two healthy controls. Polymerase chain reaction in the index case showed two different bands, a wild‐type (WT) one of approximately 700 bp and an aberrant one of approximately 500 bp not present in the controls. Subsequent Sanger sequencing showed an exon 24–26 junction, with a loss of exon 25. M, molecular weight (Hyper Ladder 100 bp, Bioline‐Meridian Neuroscience); NTC, no template control. (E) Comparison of KDM6A three‐dimensional protein structure: on the left, the WT protein; on the right, the incomplete one. Through bioinformatic tools such as ExPASy (https://www.expasy.org/) and AlphaFold Server (https://alphafoldserver.com/), we compared the canonical FASTA format of the protein with the altered one lacking exon 25, highlighting replacement of lysine for asparagine 1183 and a premature stop codon after four amino acids. (F) Ictal electroencephalographic (EEG) features (International 10–20 system, 20 s/page, low pass band filter: 70 Hz, high pass band filter: 1 Hz, gain: 150 μV/cm): epileptic spasms in drowsiness with high and diffuse diphasic slow wave and superimposed fast activity, and diamond‐like morphology of deltoid contraction. (G) Interictal EEG features (International 10–20 system, 20 s/page, low pass band filter: 70 Hz, high pass band filter: 1 Hz, gain: 150 μV/cm): bilateral independent frontotemporal spike‐and‐wave discharges in wake (phase reversal in F8 and F7, arrows).

Seizure onset was reported at 3 years, as unrecognized nocturnal epileptic spasms. The diagnosis of epilepsy was established when also diurnal clusters of spasms occurred, either spontaneous or reflex (induced by skin touch). Multiple antiseizure medications were attempted with poor efficacy (i.e., valproate, vigabatrin, rufinamide, clobazam, clonazepam). The seizure semiology was constantly stereotypical: initial fixed tonic asymmetric posturing—right arm elevation and ipsilateral head version—followed by brisk axorhizomelic adduction spasms leading to forehead banging or falls (Figure 1F). Electroencephalographic features consisted of slow background activity with bilateral independent epileptiform discharges over frontotemporal regions in wake, and spike–polyspike/wave discharges in drowsiness and sleep (Figure 1G). Brain magnetic resonance imaging was normal at age 5 years.

Whole exome sequencing (WES) on the family trio showed a novel de novo variant in *KDM6A*, c.3549‐3C>G (NM_021140.4), predicted to alter the splicing process at 3' untranslated region of exon 25 (MobiDetails, https://mobidetails.iurc.montp.inserm.fr/MD/). A similar splicing variant in *KDM6A*, c.3549‐1G>A (NM_021140.4),^5^ previously reported in association with KS type 2, and the use of phenotyping applications such as Face2Gene (https://www.face2gene.com/), shifted our focus on this condition rather than on other chromatinopathies. The c.3549‐3C>G was initially reported as a variant of uncertain origin according to the current American College of Medical Genetics and Genomics guidelines (criteria PP3 + PM1).^6^ Skipping of exon 25 observed by RNA analysis in blood (Figure 1D) allowed us to predict a premature protein truncation through bioinformatic tools (Figure 1E) and the replacement of Lys1183 in Asn in the highly conserved JmjC domain.^4^ This led us to consider the variant as likely pathogenic (PS2+ PM1)^6^ and to assess the right diagnosis of KS.

In the present case, we have recently used cenobamate as an add‐on drug to valproate/lamotrigine therapy, obtaining a reduction of spasm severity. After 6 months of treatment, seizures are limited to asymmetric tonic posturing; they occur exclusively when falling asleep and waking up. Epileptic spasms have very rarely been described in KS with *KMT2D* mutations. A general history of seizures has been reported in 36% of patients harboring *KDM6* variants,^5^ yet the specific seizure type has not yet been addressed. Epilepsy expressivity may be variable. Lederer et al.^7^ described febrile seizures only in a 3‐year‐old child, and unspecified seizures associated with a hypsarrhythmic pattern in his 6‐month‐old affected brother.^7^ Epileptic spasms have been cited in a single case in Chinese language, with no longitudinal observation.^8^ Seizures have been better characterized in other chromatinopathies. For instance, epilepsy arises with an encephalopathic phenotype that may include epileptic spasms in Wiedemann–Steiner syndrome (OMIM #605130). However, the long‐term prognosis of this type of seizure remains unknown.^9^

Our experience provides the first comprehensive description of epileptic spasms in association with alteration in the *KDM6A* gene, delving into a more precise seizure semiology and further confirming WES trio as a first‐line approach to be adopted in diagnostic studies of childhood idiopathic epilepsies, ID, and dysmorphic features.

## AUTHOR CONTRIBUTIONS

*Conceptualization:* Flavia Privitera. *Investigation:* Flavia Privitera, Camilla Meossi, Emanuele Bartolini, and Filippo Maria Santorelli. *Project administration:* Filippo Maria Santorelli. *Resources:* Flavia Privitera and Camilla Meossi. *Writing—original draft preparation:* Flavia Privitera. *Supervision:* Filippo Maria Santorelli, Emanuele Bartolini, and Camilla Meossi. *Funding acquisition:* Filippo Maria Santorelli. All authors have read and agreed to the published version of the manuscript.

## FUNDING INFORMATION

This work is supported by the Italian Ministry of Health Ricerca Corrente 2024.

## CONFLICT OF INTEREST STATEMENT

The authors declare no conflict of interest. We confirm that we have read the Journal's position on issues involved in ethical publication and affirm that this report is consistent with those guidelines.

## ETHICS STATEMENT

The study was approved by the Tuscany Region Ethics Committee (154/2020).

## INFORMED CONSENT STATEMENT

The patient gave informed consent for diagnostic testing, pictures, and research studies. The study was conducted in accordance with the Helsinki Declaration of 1964, as revised in October 2013 in Fortaleza, Brazil.

## Data Availability

Data sharing is not applicable to this article, as no datasets were generated or analyzed during the current study.
